# Successful Resolution of a Chronic Post-hysterectomy Rectocutaneous Fistula Using Transanal Minimally Invasive Surgery (TAMIS)

**DOI:** 10.7759/cureus.88832

**Published:** 2025-07-26

**Authors:** Gustavo Galicia Negrete, Alfredo S. Abarca Magallon, Daniel A. Valdivieso-Siguenza, Hector Norman Solares Sanchez, Oscar Coyoli Garcia

**Affiliations:** 1 Colorectal Surgery, Hospital Regional Licenciado Adolfo Lopez Mateos, Instituto de Seguridad y Servicios Sociales de los Trabajadores del Estado (ISSSTE), Mexico City, MEX

**Keywords:** endoluminal approach, hysterectomy complication, rectal fistula, tamis, transanal minimally invasive surgery

## Abstract

Colocutaneous fistulas are a rare but challenging complication of abdominal and pelvic surgery. Their surgical management can be difficult, especially when associated with multiple failed treatments. Transanal minimally invasive surgery (TAMIS), initially designed for local excision of rectal tumors, has been increasingly used in complex benign conditions. We present the case of a 41-year-old female patient with a chronic rectocutaneous fistula that developed following a hysterectomy and had persisted despite four failed endoscopic clip placements. The patient also reported intermittent passage of air through the vagina; however, no rectovaginal communication was identified through imaging, endoscopy, or intraoperative exploration. The fistula orifice was located 15-16 cm from the anal verge and was approached using a TAMIS platform, allowing for direct endoluminal access and precise intracorporeal suturing. The procedure lasted 65 minutes, with minimal blood loss. The patient had an uneventful recovery and was discharged 48 hours later. Long-term follow-up at three years demonstrated complete and durable resolution of the fistula, without recurrence or complications. This case illustrates the utility of TAMIS in the definitive management of complex post-hysterectomy rectal fistulas and supports its use as a safe and effective alternative to more invasive procedures in selected non-oncologic cases.

## Introduction

Enterocutaneous and colocutaneous fistulas represent one of the most serious and challenging complications in abdominal surgery, particularly when they occur as sequelae of previous surgical interventions. These lesions are defined as abnormal communications between the gastrointestinal tract, most often the colon, and the skin, resulting in the external drainage of enteric content, with significant metabolic, infectious, and nutritional consequences [1].

The global incidence of postoperative enterocutaneous fistulas varies widely, but it is estimated that 75% to 85% are secondary to abdominal surgical procedures [2]. International literature reports an incidence ranging from 0.8% to 2% following major abdominal operations [3]. However, this risk increases substantially with each subsequent reoperation: after a second laparotomy, the risk may rise to 5-10%, and in patients undergoing multiple reinterventions, rates as high as 25-30% have been described, particularly in the presence of infection, dehiscence, or manipulation of inflamed bowel loops [4].

Colonic fistulas are less frequent than those of the small intestine but are often more difficult to manage due to complex anatomy, postoperative adhesions, and microbial contamination. In Mexico, epidemiological data on enterocutaneous fistulas are scarce and primarily derived from institutional reports. Retrospective series from tertiary centers report an incidence of up to 1.5% following gynecological or complex gastrointestinal surgeries, with associated mortality rates exceeding 30% in cases of persistent sepsis or multiorgan failure [5].

Surgical management of these fistulas remains a technical and clinical challenge, especially when they originate from the distal colon or rectum. In this context, transanal minimally invasive surgery (TAMIS) has emerged as a valuable tool for evaluating and treating complex anorectal lesions [6].

## Case presentation

**Figure 1:**
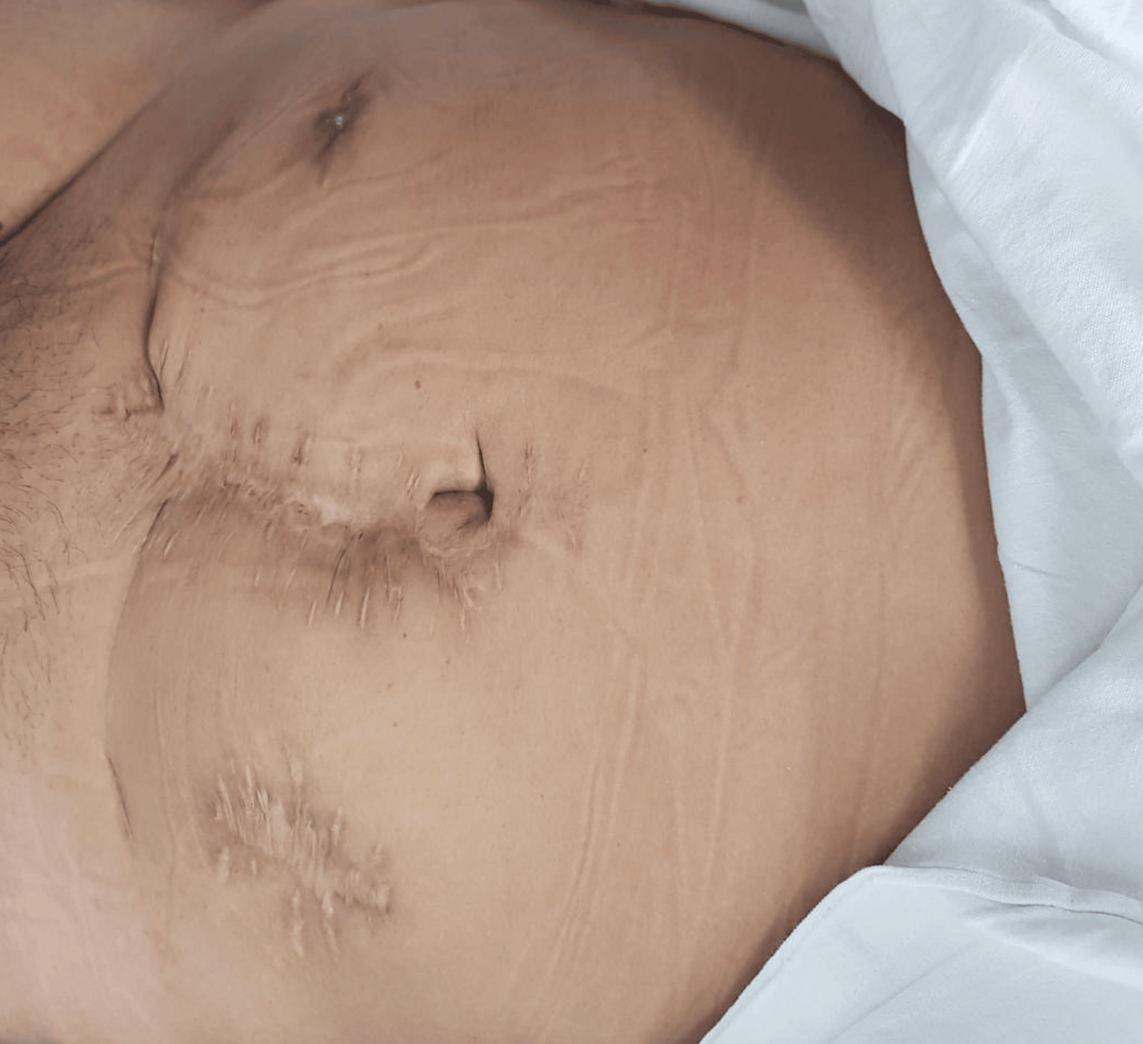
Clinical image of the lower abdomen showing a midline infraumbilical surgical scar, a healed stoma site in the left flank, and an external opening of the rectocutaneous fistula in the right iliac fossa.

**Figure 2:**
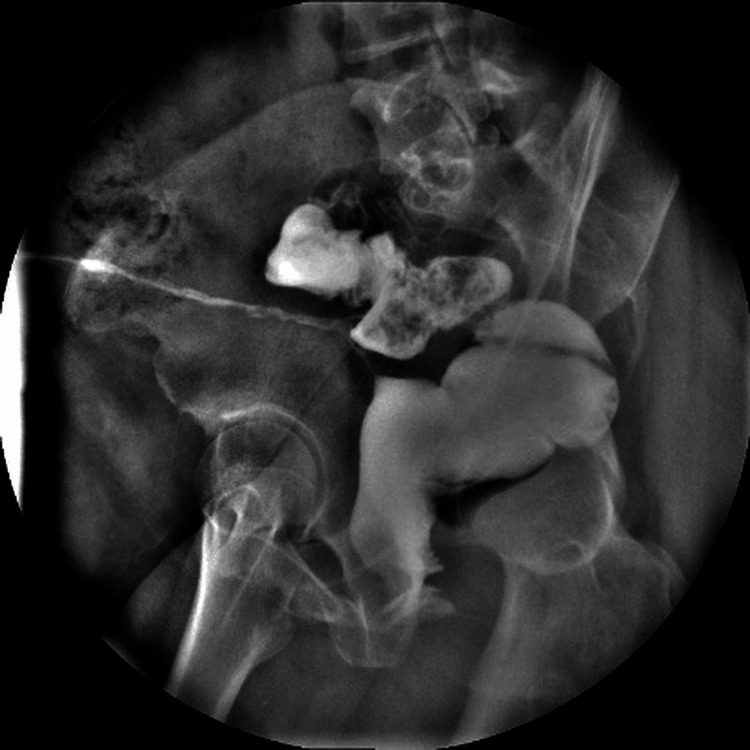
Oblique fluoroscopic view showing contrast injection into the cutaneous fistula, outlining a tract extending toward the upper rectum.

**Figure 3:**
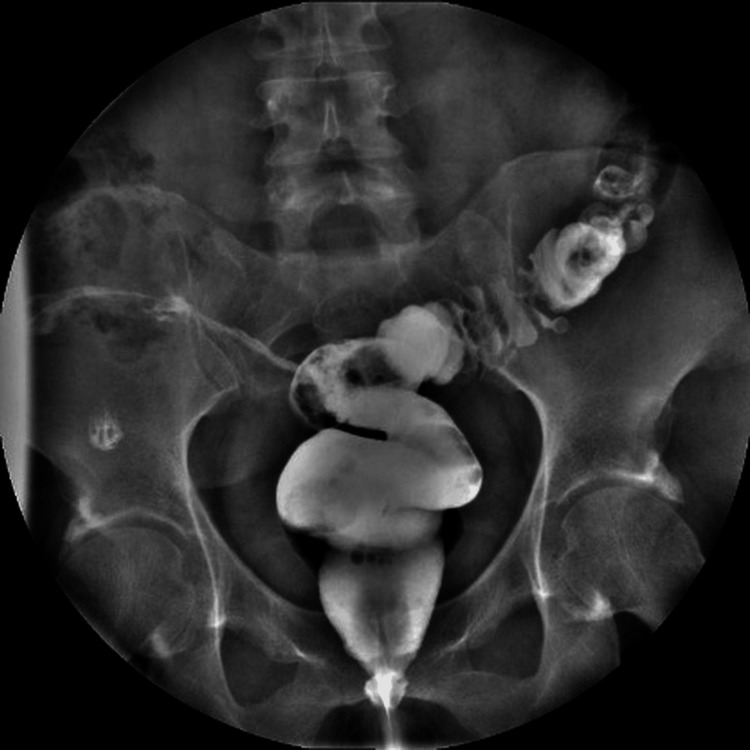
Anteroposterior fluoroscopic view demonstrating opacification of the fistulous tract and contrast passage into the upper rectum, confirming communication between skin and rectum.

A 41-year-old female patient presented with a chronic rectocutaneous fistula of four years’ duration following an abdominal hysterectomy. Her past surgical history included a right salpingo-oophorectomy 14 years prior and a hysterectomy eight years before her evaluation. She had previously undergone four unsuccessful endoscopic attempts at closure using metallic clips. The patient reported intermittent passage of air through the vagina; however, no fistulous tract to the vaginal wall was identified by any diagnostic modality. On physical examination, she exhibited a midline infraumbilical scar and a cutaneous fistulous opening in the right lower quadrant (Figure 1). Rigid rectosigmoidoscopy revealed a small internal fistulous orifice approximately 16 cm from the anal verge. Fistulography using a water-soluble contrast medium demonstrated a tract extending from the skin to the upper rectum (Figures 2, 3). Based on these findings, a minimally invasive transanal approach using the TAMIS platform was planned.

**Figure 4:**
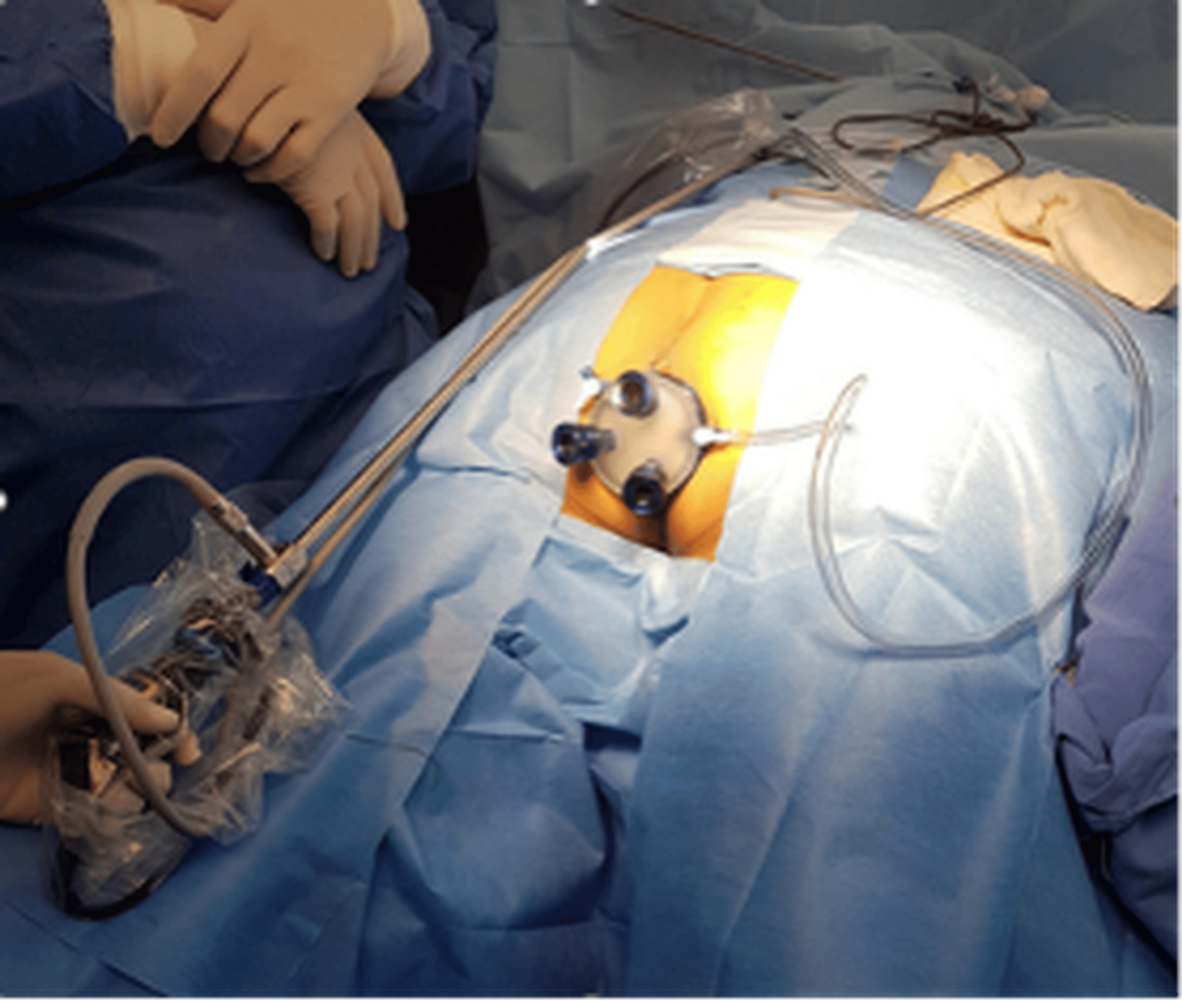
Operative view showing the patient in lithotomy position with the GelPoint Path® TAMIS platform in place, allowing transanal access with two working ports and insufflation. GelPoint Path®: Applied Medical, Rancho Santa Margarita, United States TAMIS: Transanal minimally invasive surgery

**Figure 5:**
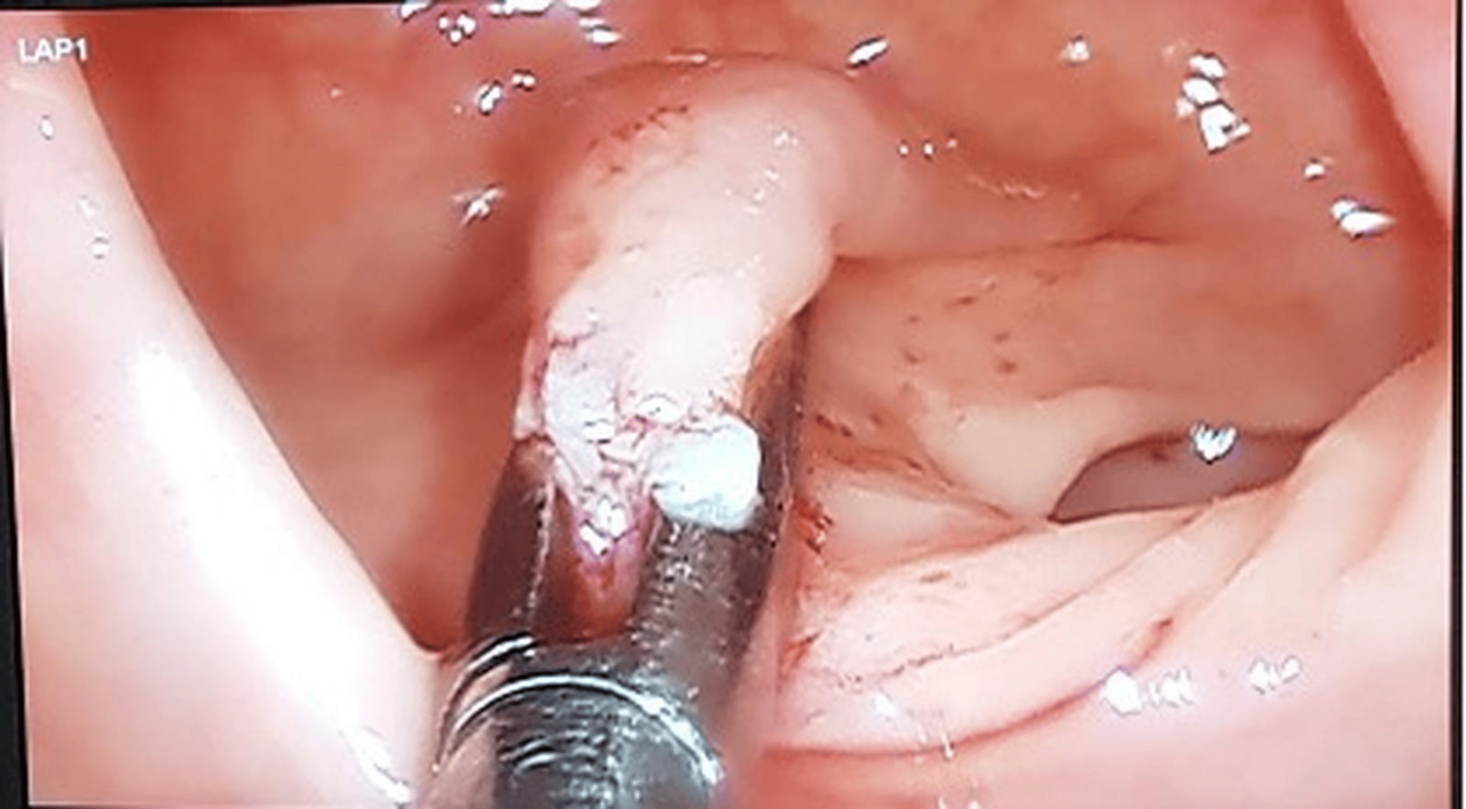
Endoluminal visualization of the rectal wall showing the internal fistulous orifice being grasped by a 5 mm atraumatic forceps.

**Figure 6:**
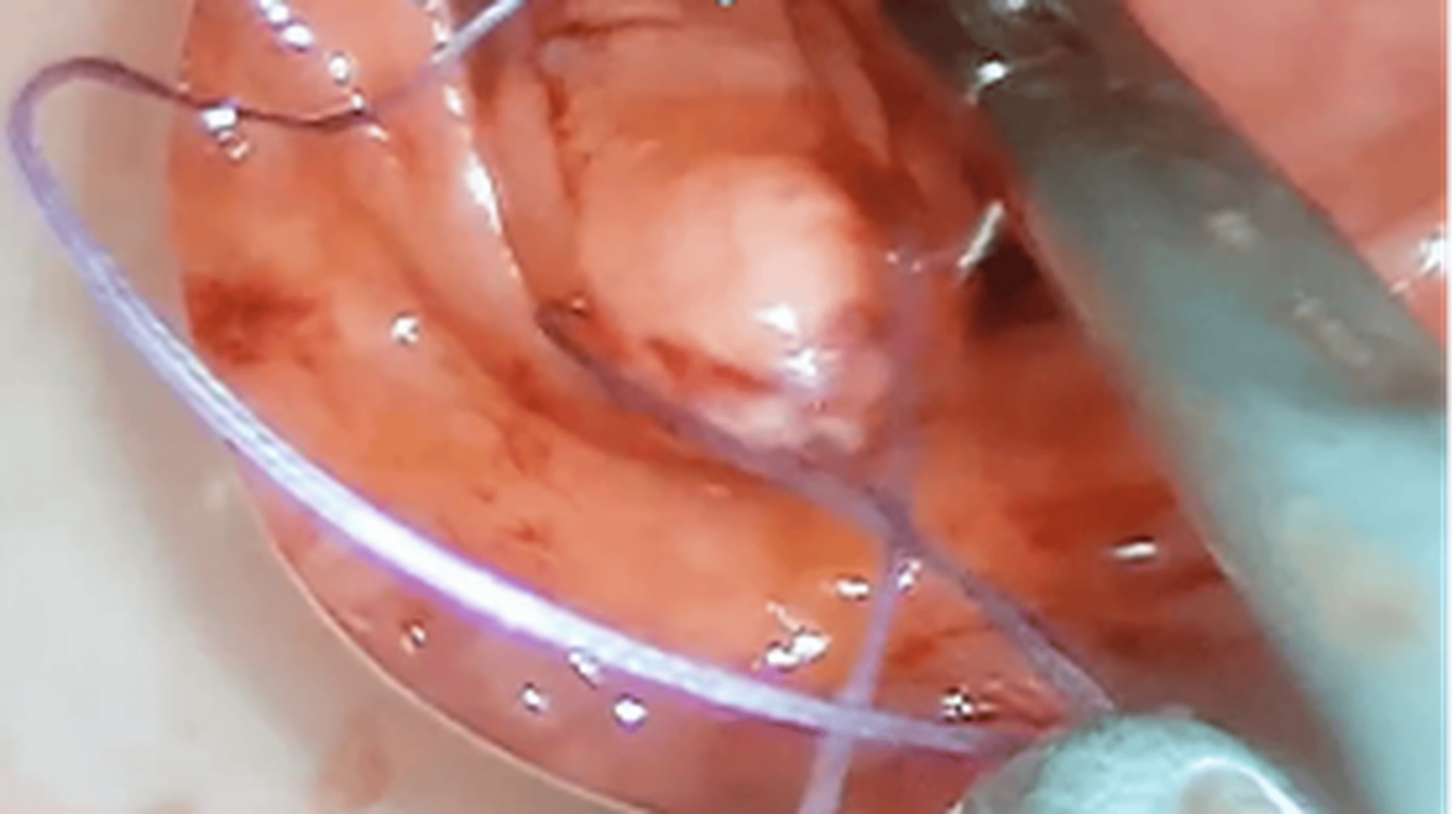
Intracorporeal closure of the fistulous tract using absorbable sutures under direct visualization through the TAMIS platform. TAMIS: Transanal minimally invasive surgery

Under balanced general anesthesia, a GelPoint Path® transanal platform (Applied Medical, Rancho Santa Margarita, United States) was inserted, allowing for endoluminal access and visualization with CO_2_ insufflation (Figure 4). The internal orifice was clearly identified at 15 cm from the anal verge. Primary closure was performed using intracorporeal absorbable sutures (Vicryl® 3-0). The procedure lasted 65 minutes with minimal blood loss (Figures 5, 6), and no intraoperative complications were observed.

Postoperative care included early enteral feeding initiated at four hours, dual intravenous antibiotic therapy, and clinical observation for 48 hours. The patient was discharged with standard hygienic and dietary recommendations and was followed regularly in the outpatient clinic. At follow-up visits on postoperative days 7 and 21, and at two, five, and nine months, and annually up to three years, she remained asymptomatic with no evidence of fistula recurrence. Complete and sustained closure of the fistula was confirmed by clinical examination.

## Discussion

TAMIS, originally developed for the local excision of early rectal tumors, has gained increasing recognition as a versatile approach for treating benign complex rectal pathologies [6]. The technique provides excellent visualization and access to the rectal lumen, allowing for precise suturing in confined pelvic spaces while avoiding the morbidity associated with open or laparoscopic abdominal approaches [7]. Although originally used in oncologic cases, several reports have demonstrated the utility of TAMIS in non-oncologic scenarios such as the closure of rectovaginal fistulas and iatrogenic rectal perforations [8,9]. These applications have expanded the indications of TAMIS, particularly in cases where conventional access is limited or failed interventions have occurred. In the present case, TAMIS was used successfully to close a chronic rectocutaneous fistula that had developed following hysterectomy and had failed multiple endoscopic treatments. The approach was safe, resulted in minimal blood loss, and achieved long-term resolution without recurrence at three years of follow-up. Understanding the pathophysiology and classification of enteric fistulas is essential to optimize treatment strategies and determine surgical timing. Chronicity, inflammation, and tissue fibrosis often complicate spontaneous healing or endoscopic closure [10]. Principles of fistula management include infection control, nutritional optimization, anatomical delineation, and definitive closure when the patient is stable and sepsis-free [11].

Recent literature supports the expansion of TAMIS applications to complex anorectal and urogenital fistulas. Marzullo et al. reported a successful TAMIS repair of a rectourethral fistula with no recurrence at 15-month follow-up, demonstrating its safety and feasibility even in urologic cases [12]. Similarly, Tobias-Machado et al. described a Brazilian case series where TAMIS was used effectively in vesicorectal fistulas, showing low morbidity and favorable recovery outcomes [13].

Compared to conventional surgical approaches, such as advancement flaps, muscle interposition, or abdominal diversion, TAMIS preserves sphincter integrity, reduces operative trauma, shortens hospitalization, and aligns with principles of enhanced recovery after surgery (ERAS) protocols [14]. Our case contributes to this growing evidence by demonstrating that TAMIS is not only feasible in post-radiation or urologic fistulas, but also in post-gynecologic surgery rectocutaneous fistulas refractory to conservative or endoscopic treatments. Long-term success in this patient reinforces the value of TAMIS as a minimally invasive and organ-preserving option in selected complex fistula cases.

## Conclusions

TAMIS represents a valuable and underutilized tool for the management of complex benign rectal conditions, including chronic rectocutaneous fistulas refractory to endoscopic closure. In this case, a long-standing colocutaneous fistula secondary to hysterectomy and multiple failed interventions was successfully managed using TAMIS, with complete healing and no recurrence after three years of follow-up. This case highlights the potential of TAMIS to offer a minimally invasive alternative to transabdominal or open surgical approaches, especially in anatomically challenging scenarios where precise access and suturing are required. The technique provides enhanced visualization of the rectal lumen and fistula orifice, facilitates a tension-free and watertight closure, and may reduce morbidity compared to traditional approaches. Its successful application in this case supports the growing role of TAMIS not only in oncologic surgery, but also in selected benign and complex pelvic pathologies where conventional options may be limited or have previously failed. Broader recognition of its benefits could expand treatment algorithms and improve outcomes in patients with difficult-to-treat anorectal fistulas.
